# Development and application of the Commitment to Profession of Medicine Scale using classical test theory and item response theory

**DOI:** 10.3325/cmj.2020.61.391

**Published:** 2020-10

**Authors:** Aysen Melek Aytug Kosan, Cetin Toraman

**Affiliations:** Department of Medical Education, Canakkale Onsekiz Mart University, Canakkale, Turkey

## Abstract

**Aim:**

To determine the level of professional commitment of medical students by developing and applying a new scale.

**Methods:**

The study enrolled 999 students of Çanakkale Onsekiz Mart University, School of Medicine. Factor analysis, reliability analysis, and item analysis were performed based on the classical test theory and item response theory. The data obtained through scale application were analyzed using factorial ANOVA.

**Results:**

The Commitment to Profession of Medicine Scale was identified as a unidimensional scale consisting of nine items. The scale in its present form explained 51% of the variance in commitment to profession of medicine. The reliability was 0.88. The scale application revealed that female students had higher commitment than male students. The highest level of commitment was observed in third- and first-year students, students with the lowest level of family income, and students whose ideal profession was medicine.

**Conclusion:**

There are many factors affecting professional commitment levels of university students. Therefore, it is of great importance to examine students' commitment at an early stage. In addition, the experiences of students during university years are important since they directly affect the commitment level. Considering these factors, teachers should support their students and strive to increase their commitment levels.

The main goal of medical education is to equip students with professional competence and prepare them for lifelong learning. Medical students should gain the motivation and skills necessary to sustain the qualifications they acquired, to acquire new qualifications, and to commit themselves to professional values (1).

Commitment to profession has been defined in several ways: as an attitude toward the profession and the work being carried out (2), as a psychological relationship between the profession and the individual, and as emotional reactions to the profession (3). Aranya et al used the term “professional bonding” (4), which relates to emphasizing identity, putting in effort in the work one performs, and adherence to professional goals, values, norms, and ethical principles (5).

Commitment of an individual to one’s job can be described as exhibiting performance in cognitive, emotional, and physical context (6-8) and emotional states creating positive excitement (9). Meyer et al identified three features of commitment to profession (10): emotional commitment, commitment to continuity, and normative commitment. Emotional commitment means being emotionally willing to pursue a profession. Individuals who are emotionally committed to their profession will have the chance to progress in their career and grow professionally. Commitment to continuity means pursuing a profession in the case of a conflict between leaving and staying. Normative commitment means that the rules of the profession have become a part of the individual’s personality. Individuals with high commitment to profession exhibit their talents at a highest level, strive to develop their career, and do not quit the profession (10,11). For this reason, it is very important to assess the level of commitment to the profession of medicine among medical students. Tools that measure commitment are available across all professions, especially in those related to educational sciences and management. However, there is no scale measuring the professional commitment to medicine. Therefore, this study aimed to develop and apply a self-report measurement tool that assesses medical school students’ commitment to the profession of medicine.

## METHODS

### Data collection instrument

A measurement tool was developed by using a previously described method (12-14). First, we identified the characteristic that was to be measured by the tool. In order to create an item pool, relevant databases were searched using the words “commitment,” “medicine commitment,” and “commitment to health” in the title or abstract. The retrieved publications were examined, and possible items were included in the scale. In addition, a focus group semi-structured interview was conducted with 10 students from different classes of the medical school. The participants provided consent for using the interview recordings. The recordings were analyzed, and possible items were included in the scale. From both sources, a pool of 38 items was created and was discussed by a panel team consisting of two medical education experts, one measurement and evaluation field expert, and two researchers. The panel agreed on a 26-item draft scale. The responses to the items were “strongly disagree,” “disagree,” “not sure,” “partly agree,” and “strongly agree.”

### Research group

After validity and reliability testing, the scale was administered to 999 students of Çanakkale Onsekiz Mart University, School of Medicine (COMUSM) on September 23, 2019. The obtained data were divided into three data sets, and each was analyzed using a different approach: 1) explanatory factor analysis (EFA), item response theory (IRT) analysis, and reliability analysis, 2) confirmatory factor analysis (CFA), and 3) scale application and comparison analyses (Table 1).

**Table 1:** Characteristics of groups of students enrolled in the study*

	No. (%) of students in		
Variables	EFA, IRT, and reliability analyses	CFA	Comparative analysis
Year			
I	47 (19)	35 (16)	93 (17.5)
II	54 (22)	41 (18.7)	110 (20.7)
III	46 (18.5)	44 (20.1)	94 (17.7)
IV	38 (15)	32 (14.6)	85 (16)
V	34 (13.7)	35 (16)	81 (15.2)
VI	29 (11.7)	32 (14.6)	69 (13)
total	248 (100)	219 (100)	532 (100)
Sex			
female	141 (56.9)	121 (55.3)	297 (55.8)
male	107 (43.1)	98 (44.7)	235 (44.2)
total	248 (100)	219 (100)	532 (100)
Family income level (€)			
0-456	104 (41.9)	84 (38.4)	218 (41)
457-913	107 (43.2)	104 (47.5)	229 (43)
914 and over	37 (14.9)	31 (14.1)	85 (16)
total	248 (100)	219 (100)	532 (100)

*EFA – explanatory factor analysis; IRT – item response theory; CFA – confirmatory factor analysis.

The minimum number of data needed for factor analysis and the number of participants in the process of developing a measurement tool was determined according to previous studies (15-20). We made certain that the study enrolled at least ten times as many participants as is the number of items in the measurement tool.

### Data analysis

The randomness of missing data was tested by using SPSS with “Estimate Mean.” The missing data were found to be random (*P* > 0.05) and, since the items in the draft scale showed a multivariate normal distribution, were completed by the mean rank of the respective variable. The suitability of the data file for factor analysis was tested with Kaiser Meyer Olkin (KMO) and Bartlett’s test of sphericity. Possible factorizations that may occur in the factor analysis were tested with Varimax rotation of the axes. Fit indices for CFA were used to assess the model fit.

The validity and reliability of the scale were assessed with IRT methods. To be able to use IRT methods for the items requiring a rated response (ie, strongly agree, partly agree, disagree, strongly disagree), unidimentionality was assessed with EFA and local independence with the Q3 statistic (21). The IRT calibrations were established using the “mirt v. 1.30” (22) package of the R v. 3.5.0 software.

## RESULTS

### Validity and reliability as assessed with CTT methods

The structure of the 26-item scale was assessed with EFA using the principal axis factoring (23). The analysis revealed a three-factor structure, but this structure was not verified by CFA.

In an effort to reduce the number of factors and find a simpler solution, the scree plot of the factor analysis was examined, and the factor analysis was reconducted with a distinct factor where the slope was the steepest. The factor analysis with a single factor showed a KMO value of 0.913 and a Bartlett’s test of sphericity value of 1538.019 (sd = 36, *P* < 0.001), which are considered satisfactory values (24,25).

**Figure 1:**
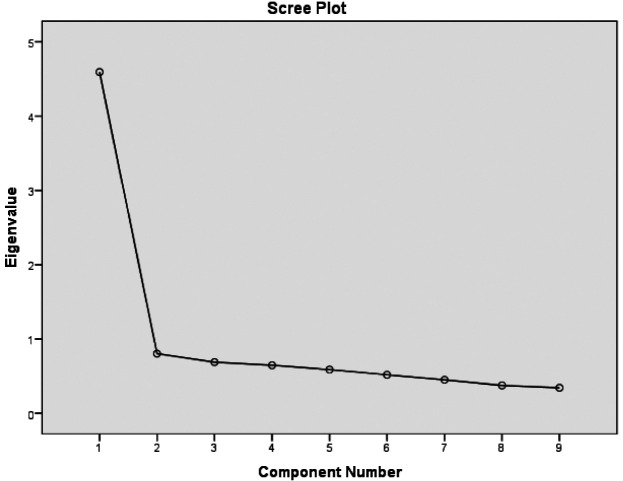
Scree plot of the nine items included in the Commitment to Profession of Medicine Scale.

In this single-factor structure, the items making the greatest contribution were 2, 4, 7, 8, 13, 16, 18, 19, and 21 (Table 2, Figure 1). The nine items exhibited a single dominant factor character with an eigenvalue of 4.592. The Cronbach alpha reliability coefficient of the nine items was 0.876 (Table 2), indicating a high level of reliability (25).

**Table 2:** Loading values of the items and total item correlations per explanatory factor analysis (EFA)

Item		Corrected item-total correlation	EFA item correlations
2	I believe that I will enjoy practicing my profession.	0.719	0.795
4	I take pride in thinking that I will become a physician.	0.581	0.678
7	Even if I face broad challenges in my profession in the future, I will still do my job with commitment.	0.693	0.778
8	No matter at what level of health care I will work as a physician in the future, I would still do my job with great devotion.	0.643	0.738
13	If I could go back to the university admission exam once more, I would still choose to become a physician.	0.553	0.650
16	I will do my best to raise quality in the profession of medicine.	0.575	0.671
18	I will do my best to raise my patients’ life quality to the highest level in the future.	0.609	0.702
19	Becoming a physician is an essential part of my whole life.	0.633	0.720
21	Spending time with patients is a great pleasure for me.	0.587	0.682
Total variance explained by the factor = 51.024			
The Cronbach Alpha reliability coefficient of 9 items = 0.876			

**Figure 2:**
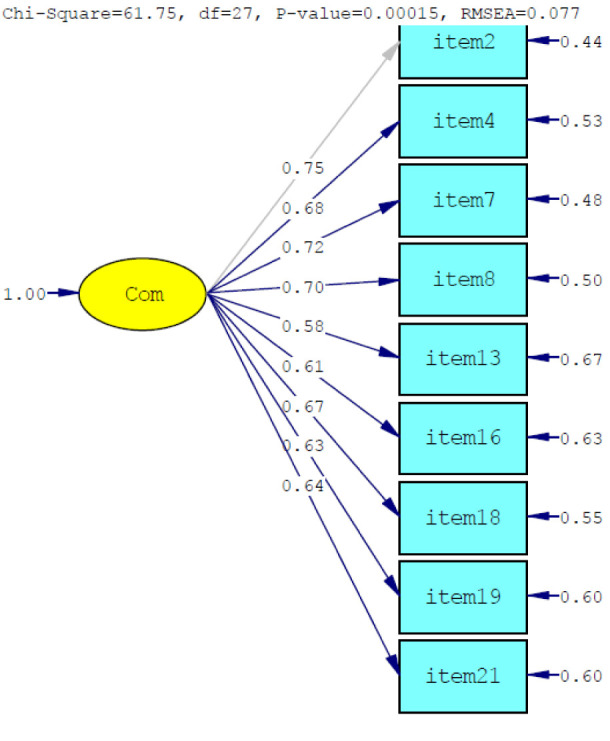
Confirmatory factor analysis results of the Commitment to Profession of Medicine Scale (standardized values). Com – medical students’ commitment to the profession of medicine.

A CFA was performed to verify the structure obtained with EFA (Table 3, Figure 2). Some of the obtained fit-indices indicated perfect fit and some indicated acceptable fit when compared with those available in the literature. This confirmed the structure obtained with EFA.

**Table 3:** Confirmatory factor analysis fit-indices for Commitment to Profession of Medicine Scale

Fit-index	Acceptable limits	Perfect fit limits	Observed value	Source
Root mean square error of approximation (RMSEA)	0.05≤RMSEA≤0.08	0≤RMSEA≤0.05	0.077	26, 27, 29
Root mean square residual (RMR)	0.05<RMR≤0.08	0≤RMR≤0.05	0.045	28, 26, 27, 15, 30
Goodness of fit index		0.90 and over	0.940	26, 15
Adjusted goodness of fit index		0.90 and over	0.900	28, 26, 15, 30
Normed fit index		0.95 and over	0.960	31, 27, 15
Incremental fit index (IFI)	0.90≤IFI≤0.94	0.95 and over	0.980	31, 27
Comparative fit index (CFI)	0.90≤CFI≤0.94	0.95 and over	0.980	31, 26, 27
X^2^/sd	2<X^2^/sd ≤5	0≤X^2^/sd ≤2	2.287	15, 25, 32

### Validity and reliability as assessed with IRT methods

The use of IRT requires unidimensionality and local independence assessment. Unidimensionality assessment with EFA showed that the scale exhibited a nine-item, unidimensional structure. Local independence was assessed with the Q3 statistic as suggested by Yen (21). Q3 showed that none of the nine items impaired local independence. Item calibrations were found for the nine items using the IRT-based generalized partial credit model (GPCM). Following GPCM, S_χ^2^, degrees of freedom, root mean square error of approximation (RMSEA), and level of significance statistics of the items were calculated (Table 4).

**Table 4:** Item response theory item-fit indices for Commitment to Profession of Medicine Scale*

Item	Generalized partial credit model			
	S_χ^2^	df	RMSEA	*P*
2	21.828	27	0.000	0.746
4	27.464	37	0.000	0.873
7	38.753	33	0.020	0.226
8	44.367	36	0.023	0.160
13	40.351	50	0.000	0.833
16	50.093	42	0.021	0.183
18	39.395	36	0.015	0.321
19	50.356	46	0.015	0.305
21	52.583	45	0.020	0.204

*RMSEA – root mean square error of approximation; df – degree of freedom; S_χ^2^ – signed χ^2^.

The RMSEA values of the items were less than 0.08, indicating a good item fit. Based on this result, it was decided that the single-factor, nine-item scale obtained with EFA satisfied the model fit as per GPCM. The “a” and “b” parameters and standard errors of the items that satisfied model fit as per GPCM were estimated (Table 5). The estimations made according to GPCM (Akaike information criterion, Bayesian information criterion, log likelihood, χ^2^, *P* < 0.001) indicated the fit of scale items.

**Table 5:** Item parameters and standard error values as per generalized partial credit model*

Item	a(SE)	b1(SE)	b2(SE)	b3(SE)	b4(SE)
2	2.076 (0.241)	-2.297 (0.300)	-2.063 (0.196)	-0.794 (0.094)	0.219 (0.082)
4	1.056 (0.126)	-2.054 (0.419)	-2.215 (0.308)	-1.279 (0.179)	-0.166 (0.127)
7	1.684 (0.186)	-1.836 (0.211)	-1.492 (0.150)	-0.167 (0.091)	1.067 (0.114)
8	1.394 (0.161)	-1.466 (0.240)	-1.658 (0.200)	-0.815 (0.124)	0.544 (0.106)
13	0.808 (0.095)	-1.693 (0.329)	-1.612 (0.258)	-0.223 (0.186)	-0.281 (0.186)
16	1.072 (0.122)	-2.476 (0.383)	-2.029 (0.240)	-0.491 (0.129)	1.102 (0.149)
18	1.264 (0.142)	-2.725 (0.427)	-2.068 (0.241)	-1.082 (0.140)	0.683 (0.115)
19	1.127 (0.125)	-1.489 (0.196)	-0.921 (0.148)	0.506 (0.131)	1.538 (0.182)
21	1.071(0.119)	-1.729(0.247)	-1.521 (0.190)	0.397 (0.129)	1.683(0.193)
Iteration = 289		Log likelihood = -4583.828		*P* < 0.001	
AIC = 9257.656		BIC = 9441.356			

*a – item discrimination; b – item difficulty; SE – standard error; AIC – Akaike information criterion; BIC – Bayesian information criterion.

**Figure 3:**
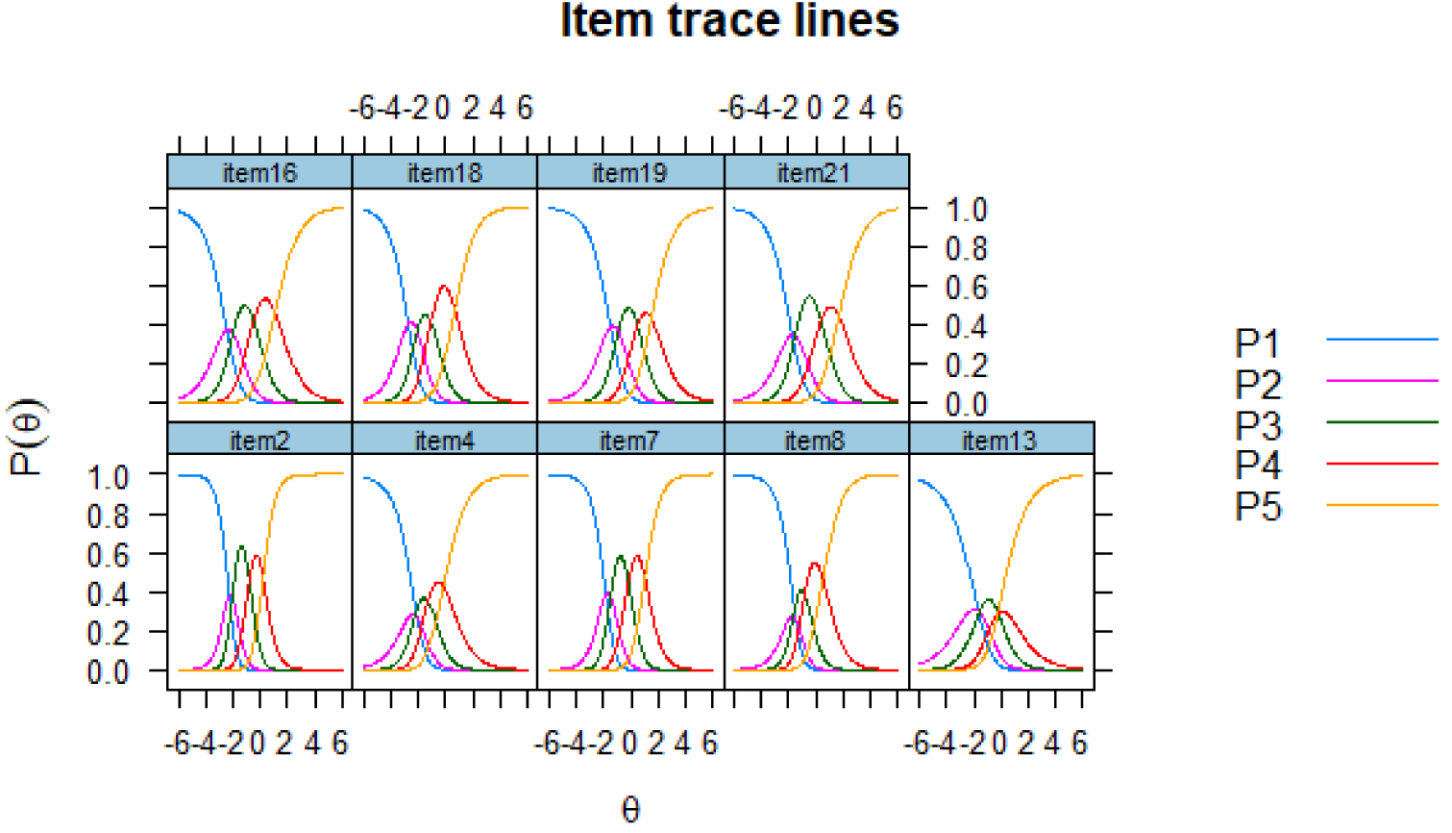
Item characteristic curves of the Commitment to Profession of Medicine Scale.

The item-characteristic curves (Figure 3) showed that the items included in the scale together with their options were significant and had a good performance for different levels of ability. The discriminative ability of the response categories of the Item 13 was relatively lower compared with the other items. The response categories of the scale items were understood by the participants and had a discriminative function.

**Figure 4:**
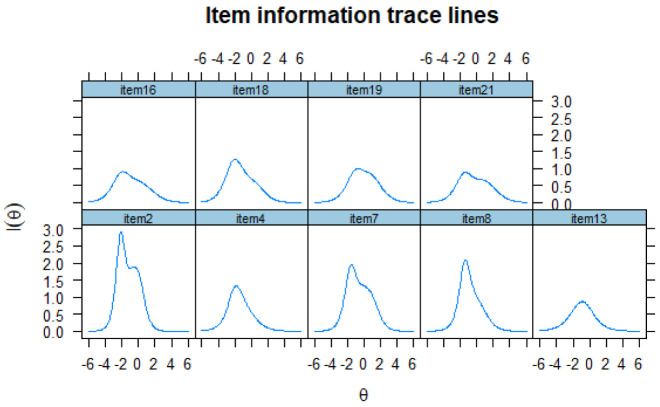
Item information functions of the Commitment to Profession of Medicine Scale.

**Figure 5:**
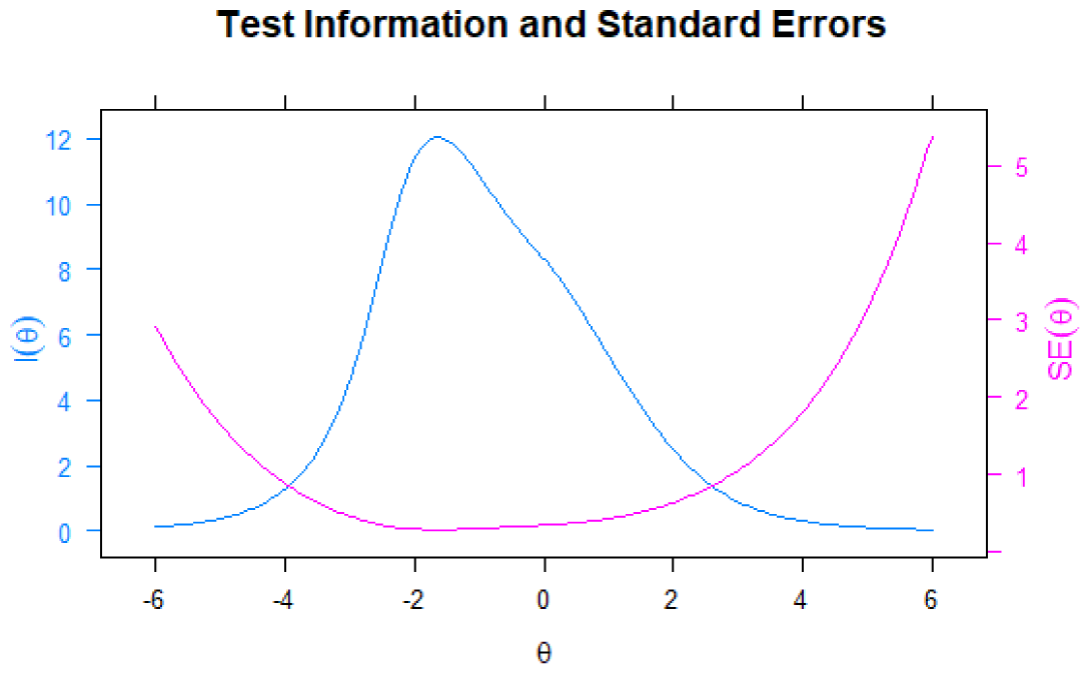
Test information function of the Commitment to Profession of Medicine Scale.

According to the item information functions, the scale items gave more information for respondents who had a low commitment level. The most informative items were 2, 7, and 8. Other items were moderately informative (Figure 4, Figure 5).

The Commitment to Profession of Medicine gives information on “professional commitment.” The scale was most informative for individuals with a commitment level in the interval between -3 and 0.5. The marginal reliability coefficient of the scale was 0.875, which is almost the same as the Cronbach alpha internal consistency value calculated according to CCT.

### The application of medical students’ Commitment to the Profession of Medicine Scale

Female students had a higher commitment level than male students (Table 6). The highest commitment level was observed among third-year students, followed by first-, second-, sixth-, fifth-, and fourth-year students. Students who had a family income between 0 and 456 € had the highest commitment level, followed by those who had a family income of 914 € and over and those who had a family income between 457 and 913 €. Students who followed medical literature besides taking notes for their lessons had a higher commitment level than those who did not. The highest commitment level was observed among the students whose “ideal profession was medicine,” followed by those who “chose medicine for other reasons,” those who “chose medicine due to its high social status,” those who “chose medicine because it is a profession with guaranteed employment and income,” and finally those who “chose medicine because their university examination score was sufficient.”

**Table 6:** Medical school students’ Commitment to the Profession of Medicine Scale (descriptive statistics)*

Variable		N	± SE mean	Mod	Median	Standard deviation	Min.	Max.
Sex	female	297	34.85 ± 0.33	35	35	5.753	19	45
	male	235	31.95 ± 0.44	30	33	6.804	9	45
Study year	I	93	34.25 ± 0.65	36	35	6.229	13	45
	II	110	33.77 ± 0.67	35	35	7.051	9	45
	III	94	35.66 ± 0.62	37	36	5.965	14	45
	IV	85	31.71 ± 0.71	32	32	6.563	9	44
	V	81	32.46 ± 0.69	32	33	6.253	20	44
	VI	69	33.10 ± 0.62	37	34	5.177	21	45
Family Income	0-456 €	218	34.65 ± 0.39	34	35	5.843	14	45
	457-913 €	229	32.62 ± 0.44	35	33	6.612	9	45
	914 € and over	85	33.38 ± 0.74	32	35	6.805	13	44
Do you follow medical literature besides than taking notes for lessons?	yes	215	34.59 ± 0.39	35	35	5.840	13	45
	no	317	32.88 ± 0.38	30	33	6.671	9	45
Why have you chosen the school of medicine?	My university examination score was sufficient for it.	95	30.35 ± 0.68	35	31	6.593	9	42
	It has a high social status.	56	32.77 ± 0.73	30	33	5.484	21	45
	It is a profession with guaranteed employment and income.	133	30.54 ± 0.53	30	30	6.148	9	44
	It is the only ideal profession for me.	211	37.26 ± 0.32	36	38	4.621	13	45
	Other	37	32.95 ± 1.02	33	34	6.231	20	45

*N – number of students; – mean; SE – standard error; Min. – minimum score; Max. – maximum score.

The levels of commitment to profession with respect to students’ sex, study year, family income, following medical literature besides taking notes for their lessons, and the reason for choosing medicine were compared using the factorial ANOVA method with Bonferroni *post-hoc* test (Table 7, Table 8).

**Table 7:** Medical school students’ commitment to profession of medicine with respect to sex, class, and family income (factorial ANOVA)*

Source of variance	Sum of squares	df	Mean square	F	*P*	η^2^
Sex	866.387	1	866.387	24.529	0.001	0.047
Class (2019-2020, year I-VI)	985.724	5	197.145	5.582	0.001	0.053
Family income	268.146	2	134.073	3.796	0.023	0.015
Sex × class	196.254	5	39.251	1.111	0.353	0.011
Sex × family income	6.205	2	3.103	0.088	0.916	0.000
Class (2019-2020, year I-VI) * family income	792.810	10	79.281	2.245	0.014	0.043
Sex × class × family income	161.996	10	16.200	0.459	0.719	0.000
Error	17519.059	496	35.321			
Total	621320.000	532				

*df – degree of freedom; F – F test with ANOVA; *P* – significance; η^2^ – eta square effect size.

**Table 8:** Medical school students’ commitment to profession of medicine with respect to following medical publications besides taking notes for their lessons and the reason for studying medicine (factorial ANOVA)*

Source of variance	Sum of squares	df	Mean square	F	*P*	η^2^
Following medical publications other than taking notes for lessons	78.071	1	78.071	2.499	0.115	0.005
Reasons for choosing school of medicine	4496.501	4	1124.125	35.978	0.001	0.216
Following medical publications other than taking notes for lessons * Reasons for choosing school of medicine	204.705	4	51.176	1.638	0.163	0.012
Error	16309.719	522	31.245			
Total	621320.000	532				

*df – degree of freedom; F – F test with ANOVA; *P* – significance; η^2^ – eta square effect size.

Female students had a significantly higher commitment (F_(1-496)_ = 24.529, *P* < 0.05), with a moderate effect size (η^2^ = 0.05) (33). Commitment to profession significantly differed according to the study year (F_(5-496)_ = 5.582, *P* < 0.05), with a moderate effect size (η^2^ = 0.05). First-year and third-year students had higher commitment to profession than fourth-year students. Third-year students had higher commitment to profession than fifth-year students. Students whose family income was between 0 and 456 € had a significantly higher commitment to profession than those whose family income was between 457 and 913 € (F_(2-496)_ = 3.796, p≤0.05), with a small effect size (η^2^ = 0.02).

The interaction of the basic effects of study year and family income created a significant difference in commitment to profession (F_(10-496)_ = 2.245, *P* < 0.05), with a moderate effect size (η^2^ = 0.04). First-year students with family income of 0-456 € had a higher commitment level than second-year students with family income of 457-913 €. Second-year students with family income of 0-456 € had a higher commitment level than second-year students with family income of 457-913 €.

Commitment to profession significantly differed among students with different reasons for studying medicine (F_(4-522)_ = 35.978, *P* < 0.05), with a large effect size (η^2^ = 0.22). Students who reported that medicine was their ideal profession had higher commitment than students who listed other reasons for studying medicine.

Commitment to profession did not significantly differ among students who followed medical literature besides taking notes for lessons, and the interaction of this variable with the reasons for studying medicine did not create a significant difference (*P* > 0.05).

## DISCUSSION

In this study, using both CTT and IRT, we developed a one-dimensional, nine-item Commitment to Medical Profession Scale for university students. The scale application showed that female students had higher commitment levels than male students. Higher commitment levels in women were also observed in studies among nurses (34,35) and teachers (36), the latter showing that women were more committed than male teachers in all domains except commitment to work group. Contrary to this, higher commitment levels among men were found among students (37), administrative staff of a state university (38), and nurses (39). In some studies, commitment level did not significantly differ between the sexes (40,41).

Another important result of our study was the relationship between the professional commitment levels and family income. Namely, students with low or high family income were more committed to profession than students with moderate family income. Similarly, Saruc et al (41) showed that the professional commitment level of social workers increased with the increase in income level.

We also observed higher professional commitment of students who followed scientific literature besides taking course notes. These findings show that students who develop their professional skills were more committed to their profession. Since best practices and patient care standards are constantly evolving, physicians are expected to follow the recent developments in the field (42,43) and undergo continuous training in order to provide the best care for the patients. Within this context, another study reported that nurses believed that continuing education was as an investment into specialization in the field (44). Considering all this, students should be encouraged to get involved in professional development events.

Another important finding was that professional commitment level of first-year and third-year students was higher than that of fourth-year students. In addition, the commitment level of third-year students was significantly higher than that of fifth-year students. Therefore, the students’ commitment level decreased during the course of the studies. In other words, it was negatively affected by students’ experiences during university education. These experiences could include formal internships, jobs, and social work, as well as mentoring and counselling they received (45).

Students who stated that they chose this profession because it was their ideal job had higher commitment level. This indicates that choosing a profession based on students’ life goals is more important than basing this choice on other factors. Moreover, career choice is one of the biggest dilemmas and challenges in students’ lives (46) and is affected by many factors, such as family, passion, salary, and experiences (47). In other studies, students reported their career choice to be significantly affected by salary (48,49) but also by cultural factors, career aims, professional status, as well as family factors (50).

A strength of this study is a high participation rate – only 11 (1%) students refused to participate. Despite the high number of participants, the fact that the study was carried out in only one medical school in Turkey limits its generalizability. In order to ensure that the validity and reliability of the scale did not depend on the group, IRT methods were applied alongside with CTT methods. On the other hand, repeating the validity and reliability assessment on data obtained from different groups can contribute to the development of the scale.

In conclusion, the results of this study revealed different factors affecting professional commitment levels of university students, the most significant of which were sex, family income, attending professional development events, following the recent developments in the field of medicine, a high social status of medicine, and salary. Therefore, it is important to evaluate students' professional commitment level at an early stage. In addition, universities should create opportunities for students to take part in experiences that positively affect their commitment, such as professional development trainings. Teachers should also strive to support students in their professional development and enable access to medical literature.
